# Adenoma detection in patients undergoing a comprehensive colonoscopy screening

**DOI:** 10.1002/cam4.73

**Published:** 2013-04-20

**Authors:** Gottumukkala S Raju, Vikram Vadyala, Rebecca Slack, Somashekar G Krishna, William A Ross, Patrick M Lynch, Robert S Bresalier, Ernest Hawk, John R Stroehlein

**Affiliations:** Department of Gastroenterology, Hepatology and Nutrition, University of Texas MD Anderson Cancer CenterHouston, Texas, 77030

**Keywords:** Adenoma, colon, colonoscopy, detection, serrated adenoma

## Abstract

Measures shown to improve the adenoma detection during colonoscopy (excellent bowel preparation, cecal intubation, cap fitted colonoscope to examine behind folds, patient position change to optimize colon distention, trained endoscopy team focusing on detection of subtle flat lesions, and incorporation of optimum endoscopic examination with adequate withdrawal time) are applicable to clinical practice and, if incorporated are projected to facilitate comprehensive colonoscopy screening program for colon cancer prevention. To determine adenoma and serrated polyp detection rate under conditions designed to optimize quality parameters for comprehensive screening colonoscopy. Retrospective analysis of data obtained from a comprehensive colon cancer screening program designed to optimize quality parameters. Academic medical center. Three hundred and forty-three patients between the ages of 50 years and 75 years who underwent first screening colonoscopy between 2009 and 2011 among 535 consecutive patients undergoing colonoscopy. Comprehensive colonoscopy screening program was utilized to screen all patients. Cecal intubation was successful in 98.8% of patients. The Boston Bowel Preparation Scale for quality of colonoscopy was 8.97 (95% confidence interval [CI]; 8.94, 9.00). The rate of adenoma detection was 60% and serrated lesion (defined as serrated adenomas or hyperplastic polyps proximal to the splenic flexure) detection was 23%. The rate of precancerous lesion detection (adenomas and serrated lesions) was 66%. The mean number of adenomas per screening procedure was 1.4 (1.2, 1.6) and the mean number of precancerous lesions (adenomas or serrated lesions) per screening procedure was 1.6 (1.4, 1.8). Retrospective study and single endoscopist experience. A comprehensive colonoscopy screening program results in high-quality screening with high detection of adenomas, advanced adenomas, serrated adenomas, and multiple adenomas.

## Introduction

Colonoscopic removal of adenomatous colon polyps has been shown to reduce the incidence of colorectal cancer and also to prevent death from colorectal cancer [1, 2]. Although colonoscopy is widely used for colorectal cancer screening, its miss rate for cancer and that for adenomas remains a concern [3–5]. The adenoma detection rate is an independent predictor of the risk of interval cancer and the ability of colonoscopy to reduce the incidence of colorectal cancer depends on the removal of adenomas [1, 6]. Recently, measurement of adenoma detection has been included in quality-improvement programs for colorectal cancer screening [7, 8]. The US Multi-Society Task Force on Colorectal Cancer has established target adenoma detection rates of >25% for men, and >15% for women undergoing screening colonoscopy [9]. Recently, the NHS Bowel Cancer Screening Program in the United Kingdom recommended target adenoma detection rate of ≥40% and mean adenoma detection per patient of 1.20 in patients [10].

The National Polyp Study predicted reduction in the incidence of colorectal cancer and colorectal cancer deaths in an adenoma-bearing cohort that had undergone clearing colonoscopy [1, 2]. However, recent dietary and chemoprevention trials suggest a lower level of protection [3, 11, 12]. Potential causes for colonoscopy failure to prevent colorectal cancer include the following: (a) inadequate bowel preparation [13–16], (b) failure to reach cecum [17, 18], (c) abbreviated withdrawal of colonoscope [19], (d) failure to examine polyps hidden behind folds [20–23], (e) inability to recognize subtle flat lesions such as sessile serrated adenomas [24–26], (f) incomplete polypectomy [5], (g) failure to provide interval surveillance colonoscopy program in patients with adenomas [27], and (g) biological differences in colorectal neoplasia growth [28].

From the standpoint of the practicing endoscopist who performs high-quality screening colonoscopy, a number of measures are applicable to clinical practice in order to (a) provide for comprehensive colonoscopy screening and (b) improve the quality of the procedure [29, 30]. These include achieving excellent quality of bowel preparation using a split dose bowel preparation [31–35], reaching the cecum to screen the entire length of the colon [18], taking adequate time to examine the colon [19], using a good technique to circumferentially examine a distended colon with a cap fitted colonoscope [36–39], using a high-definition endoscope with a trained eye to detect subtle flat lesions [24, 40], and reporting the colonoscopy findings using a quality structured reports [41].

To maximize the effectiveness of colonoscopy as a prevention tool, an endoscopist (G. S. R.) incorporated multiple quality measures into clinical practice to develop a comprehensive colonoscopy screening program between 2001 and 2008 [42]. Although an expert panel recognized the importance of detection of serrated lesions, it is not clear whether this comprehensive colonoscopy screening program has an impact on serrated lesion detection [43]. Analysis of the data from this experience was undertaken to determine the adenoma detection and serrated lesion detection rate in a cohort of 343 consecutive patients between the ages of 50 years and 75 years who had undergone their first screening colonoscopy using comprehensive colonoscopy screening program by a single endoscopist (G. S. R.) between 2009 and 2011 at a single cancer center.

## Material and Methods

### Patients

All patients referred for their first screening colonoscopy between June 2009 and December 2011 who did not have a family or personal history of polyposis, inflammatory bowel disease, or a personal history of prior polypectomy or colorectal cancer were included in the retrospective study. The Institutional Review Board at the University of Texas MD Anderson Cancer Center approved the study. A single endoscopist (G. S. R.) performed all the procedures using a comprehensive colonoscopy screening program under conscious sedation (Table 1).

**Table 1:** Comprehensive colonoscopy screening program

Colon preparation	Split dose of polyethylene glycol electrolyte solution and bisacodyl in divided doses [32] (see Video S1)
Colonoscope	Cap fitted colonoscope [38]
Cleaning on insertion	Simethicone solution flush followed by suction to achieve BBPS of three in each segment before endoscope insertion into the cecum [Bibr b34], [35]
Endoscope insertion	Colonoscope insertion to the cecum at 60–80 cm
Endoscopic screening	Cap fitted endoscope to examine in between folds [38]
	Patient position change to optimize colon distension [37]
	Circumferential scanning for subtle flat lesions of the colon by focusing on disturbance to the mucosal innominate groove pattern, surface pit pattern, and vascular architecture [36]
	Training the eyes by reviewing the videos and photos of subtle lesions and the endoscopy team focus on finding subtle lesions [47, 48]
Endoscopic report	Standardized colonoscopy reporting and data system for comprehensive recording of the procedure details [49].
Follow-up	Counsel about pathology findings and surveillance colonoscopy intervals

BBPS, Boston Bowel Preparation Scale.

### Comprehensive colonoscopy screening program

The comprehensive colonoscopy screening program is summarized in Table 1.

### Intensive bowel preparation regimen for colon preparation

All patients were instructed about colon preparation and were encouraged to view patient education videos developed of our institution on the YouTube (see Video S1). The protocol included six bisacodyl tablets in divided doses and 4 L of polyethylene glycol (PEG)-based electrolyte solution in a split dose. It consisted of a light breakfast and lunch without vegetables, salads, fruits and nuts, two bisacodyl tablets (5 mg) at noon, followed by another dose at 3.30 pm and 2 L of PEG solution on the day before the procedure. Those scheduled to have a procedure before noon were instructed to take two tablets of bisacodyl and 2 L of the PEG solution in the early morning 3 am, while those scheduled to have a procedure in the afternoon were instructed to take two tablets of bisacodyl and 2 L of the PEG solution at 7 am [32].

### Colonoscopy screening

#### Setup of video monitor

The video monitor was setup about 4–5 feet from the endoscopist at or below the endoscopist's eye level [44]; usually this amounts to keeping the monitor above the bed rail on the other side of the patient.

#### Cap fitted colonoscopy

Either a cap fitted pediatric or adult variable stiffness colonoscope of the Olympus 160 series, with a fixed 140° angle of view was used for screening. The cap extended 2–3 mm from the tip of the endoscope [38]. Carbon dioxide was used only if the intubation through the sigmoid colon became technically challenging and if the patient required endoscopic mucosal resection. Conscious sedation was used for the procedures.

#### Clean and dry the colon during colonoscope insertion

During the endoscope insertion, simethicone mixed water was routinely flushed in the left, transverse, and right colon and all the fluid was aspirated to create a dry colon before endoscope insertion to the cecum so that on withdrawal a clean dry colon (Boston Bowel Preparation Scale of 3 of 3 in each segment) was encountered for examination without the need for additional water flushes and suction [34, 35].

#### Cecal insertion without loops

Every attempt was made to reach the cecum, unless the endoscopist felt that it was risky and futile to pursue further. The colon loops were reduced constantly during the endoscope insertion so that the cecum could ideally be reached at 60–80 cm. A short straight endoscope allowed controlled withdrawal of the endoscope and circumferential scanning of the colon.

#### Screening – adequate distension of the colon

The patient's position was changed from left lateral to supine or right lateral position to allow adequate distension of the colon [37].

#### Screening – working the folds to pick up polyps behind folds

The colonoscopic screening involved working the folds with the cap to examine behind the ileo-cecal valve and in between the haustral folds. The cap was pushed against the colon wall to open up the mucosa if the folds were crowded [36].

#### Screening for subtle lesions – training the eye for subtle lesions

The endoscopist trained his eyes to detect subtle lesions initially by looking at the endoscopy atlases and videos on the subject [45–47], working closely with an expert in the field sharing and reviewing videos of subtle flat lesions on the web and at the American Society Gastrointestinal Endoscopy Courses on Colonoscopy. The endoscopist (G. S. R.) trained his endoscopy technicians by reviewing photos and videos of subtle lesions [47].

#### Screening for subtle lesions – technique to look for subtle lesions

The endoscope was withdrawn by keeping the endoscope close to the wall, as the endoscope worked the folds circumferentially and with to-and-fro movements, so that the subtle changes in the architecture of the innominate grooves, mucosal vasculature, and surface pit pattern could be closely observed with white light to identify subtle lesions [36]. Dye spray chromoendoscopy and narrow band imaging were not used to detect lesions. If any mucus was encountered, it was washed and the area was closely examined for serrated polyps. When a second pair of eyes (trained endoscopy technicians and nurses) spotted a lesion, that area was closely examined. Once all members of the team agreed about the presence or absence of a finding and it was appropriately dealt with, the endoscopist continued with examination of the rest of the colon. Video recording of all subtle lesions and all large lesion resections were routinely undertaken. Photos and videos were reviewed with the team to educate the team in finding subtle lesions. If polyps were found during insertion, they were removed at that time [48].

#### Screening time

The goal was to screen the colon and whatever time was required to screen the entire colon depending on the individual anatomy for any subtle lesion (see Video S2) was taken (a minimum of 6 min of screening time is required as part of the quality initiative of our endoscopy unit). The endoscopy unit at our institution allowed scheduling an hour for colonoscopies to deal with removal of all polyps, including large and flat lesion resections, and completion of a detailed procedure note and counseling of the patients and family about the findings of the procedure.

#### Polyp removal

Cold biopsy was used to remove smaller lesions (<5 mm) and endoscopic mucosal resection was used for lesions larger than 5 mm. All the lesions, including the large ones were removed during the same session. Each polyp was submitted in a separate jar with accurate labeling of the jar to correspond with the polyp removed and documented in the endoscopy report for pathological correlation and assessment of polyp burden.

### Endoscopy report

Based on the recommendations of the Quality Assurance Task Group of the National Colorectal Cancer Roundtable [49], we developed a standardized colonoscopy reporting system (Endoworks, Olympus Inc., Center Valley, PA) that provided for data entry and patient report generation. Two to three photos of the appendicular orifice, cecum, and ileocecal valve were taken to document cecal insertion. The Boston Bowel Preparation Scale was used to define the quality of colon preparation; photos were taken to document the quality of preparation [35]. Each polyp was submitted in a separate jar for pathology to help in the counting of the polyp burden.

### Pathology of polyps

One of our dedicated gastrointestinal pathologists reviewed the pathology slides and reported the findings using standard terminology, including a comment on the completeness of resection of the large polyps.

#### Follow-up

The endoscopist contacted all the patients by phone 1–2 weeks after the procedure, followed by a letter that included counseling about biopsy findings and recommendations of surveillance examination based on the American Society of Gastrointestinal Endoscopy (ASGE) guidelines. Patients with no adenomas during the screening colonoscopy were recommended to reevaluate their risk for colon cancer at 5 years based on their family history and personal health and risk factors.

#### Data-collection and statistical analysis

The patients' demographics, medical, surgical, and cancer illnesses were retrieved from the hospital electronic medical record and the endoscopy data were retrieved from the colonoscopy report database and entered into an ACCESS Colonoscopy Database.

Nearly half of the patients in our cohort had a history of cancer (for example, breast cancer, prostate cancer, skin cancer, etc.). To evaluate the impact of cancer history on adenoma detection, the cohort was divided into those with history of any cancer “the cancer group” and those with no history of cancer, “the non-cancer group” and the results in both groups were compared.

### Statistical analysis

Patients were selected from the overall screening colonoscopy database to meet the criteria of having their first screening colonoscopy with average risk between the ages of 50 years and 75 years. Patient characteristics and colonoscopy elements were tabulated overall and by cancer history. Colonoscopy experience was described by time in minutes and successful insertion and preparation. Among this defined population, each polyp found was classified as one of the following: cancer, precancerous (adenoma, serrated polyp, or both), or other (including non-neoplastic lesions and distal hyperplastic polyps). Specific definitions for detection rates and polyp burden are detailed in Table 2. Polyp counts for each type were summarized with counts and proportion for each patient and overall. Histograms of polyp counts were created overall and for men and women separately. The overall distribution of polyp types by cancer history was tested by the Jonkheere-Terpstra test as implemented in StatXact v8 (Cytel Inc., Cambridge, MA) to account for the natural orderings of polyp severity and cancer history. For individual comparisons of proportions, chi-square tests were implemented. For continuous measures, mean and 95% CI were reported. Comparisons between means were made with *t*-tests. All data analyses were performed in SAS 9.2 (SAS Institute, Inc., Cary, NC) unless otherwise noted. Plots were created in Stata/SE 12.1 (StataCorp LP, College Station, TX).

**Table 2:** Definitions of adenoma detection rate and adenoma burden

Adenoma detection rate	
Adenoma detection rate	Percentage of patients with tubular, tubullovillous, or villous adenomas
Advanced adenoma detection rate	Percentage of patients with advanced adenomas (>1 cm in size, villous histology, high-grade dysplasia)
Multiple adenoma detection rate	Percentage of patients with ≥3 adenomas
Serrated lesion detection rate	Percentage of patients with serrated adenomas or hyperplastic polyps proximal to splenic flexure
Precancerous lesion detection rate	
Precancerous lesion detection rate	Percentage of patients with adenomas or serrated lesions
Adenoma burden	
Mean number of adenomas per screening procedure	Total number of adenomas detected divided by the number of screening procedures
Mean number of adenomas per screening procedure positive for precancerous lesions	Total number of adenomas detected divided by the number of screening procedures positive for precancerous lesions

Precancerous lesions include adenoma and serrated lesions.

## Results

Three hundred forty-three patients who underwent screening colonoscopy between the ages of 50 years and 75 years by a single endoscopist, at the University of Texas, MD Anderson Cancer Center, between 2009 and 2011, were included in the study. In one patient, data on cancer history were missing. The remaining 342 patients were divided into two groups: (a) No cancer history group (*n* = 179) consisted of patients with no history of cancer, and (b). Cancer group (*n* = 163) consisted of patients with cancer history other than colorectal cancer.

### Demographics

The patient characteristics are presented in Table 3 overall and by cancer history. The mean age of the 343 patients was 58.2 years, with 134 men and 209 women. The majority of the patients were Caucasians (71%), with a history of prior non-colon cancer in 163 (48%), comorbid medical conditions in 209 (61%), and a median body mass index (BMI) ranging from 15 to 59).

**Table 3:** Patient characteristics overall and by cancer history

	All patients	Patients without cancer history	Patients with cancer history
Characteristic	*N* (%)	*N* (%)	*N* (%)
All patients	343 (100)	179 (100)	163 (100)
Age in years (median 57)			
50–59	213 (62)	128 (72)	85 (52)
60–69	108 (31)	48 (27)	59 (36)
70–75	22 (6)	3 (2)	19 (12)
Gender			
Female	209 (61)	107 (60)	102 (63)
Male	134 (39)	72 (40)	61 (37)
Race			
Caucasian	242 (71)	124 (69)	117 (72)
African American	40 (12)	21 (12)	19 (12)
Asian	39 (11)	26 (15)	13 (8)
Hispanic	20 (6)	6 (3)	14 (9)
Other	2 (1)	2 (1)	0 (0)
Body mass index			
Underweight	7 (2)	3 (2)	4 (2)
Normal weight	89 (26)	43 (24)	46 (28)
Overweight	135 (39)	76 (42)	59 (36)
Obese	96 (28)	44 (25)	51 (31)
Missing	16 (5)	13 (7)	3 (2)
Smoking status			
Yes	23 (7)	10 (6)	13 (8)
Former	65 (19)	29 (16)	36 (22)
No	255 (74)	140 (78)	114 (70)
Comorbid conditions			
Yes	209 (61)	97 (54)	112 (69)
No	124 (36)	79 (44)	45 (28)
Missing	10 (3)	3 (2)	6 (4)
Cancer history			
Yes	163 (48)	0 (0)	163 (100)
No	179 (52)	179 (100)	0 (0)
Missing	1 (0)	–	–

There was no difference between the cancer (163) and no cancer (179) groups except for the older age of patients with means of 60.0 (58.9, 61.0) and 56.5 (55.8, 57.2) years, respectively (*P* < 0.001), and comorbid conditions (69% vs. 54%, *P* = 0.01) in the cancer group compared to the no cancer group.

### Colonoscopy

Patients primarily received fentanyl and midozolam (83%) with the pediatric size colonoscope (96%). Procedure success and time measures are presented in Table 4. Cecal intubation was successful 98.8% of patients. The Boston Bowel Preparation Scale for quality of colonoscopy was 8.97 (95% CI 8.94, 9.00). Average cecal insertion time and total procedure time were 12.0 and 41.0, respectively. There was no difference between the cancer and no cancer groups in cecal insertion, quality of colonoscopy preparation, cecal insertion time, and total procedure time.

**Table 4:** Colonoscopy findings in 343 consecutive patients between the ages of 50 years and 75 years undergoing first screening colonoscopy

Characteristic	All patients	No cancer	Cancer
All patients N*	343	179	163
Cecal insertion – successful *N* (%)	339 (98.8%)	179 (100%)	159 (97.6%)
Cecal insertion time in minutes mean (95% confidence interval)	12.0 (11.2, 12.7) *N* = 315	11.8 (10.7, 12.9) *N* = 166	12.2 (11.1, 13.3) *N* = 148
Total procedure time in minutes mean (95% confidence interval)	41.0 (39.7, 42.4) *N* = 322	41.2 (39.4, 43.0) *N* = 167	40.8 (38.7, 42.8) *N* = 154
Boston Bowel Preparation Scale (0–9) mean (95% confidence interval)	8.97 (8.94, 9.00)	8.98 (8.95, 9.00)	8.96 (8.91, 9.00)

* Measures that have *N* specified were not available for all patients, otherwise all patients were included in the calculation.

Average cecal insertion and total colonoscopy procedure times in patients with no polyps were 12.8 (10.2, 15.5) min and 36.0 (32.1, 39.8) min, respectively, while the average cecal insertion and total colonoscopy procedure times in patients with polyps was 11.8 (11.0, 12.7) min and 42.0 (40.3, 43.2) min, respectively. The overall procedure was on average 5.8 (1.7, 9.9) min longer among patients with polyps (*P* = 0.01).

### Polyp characteristics and detection

**Figure 1:**
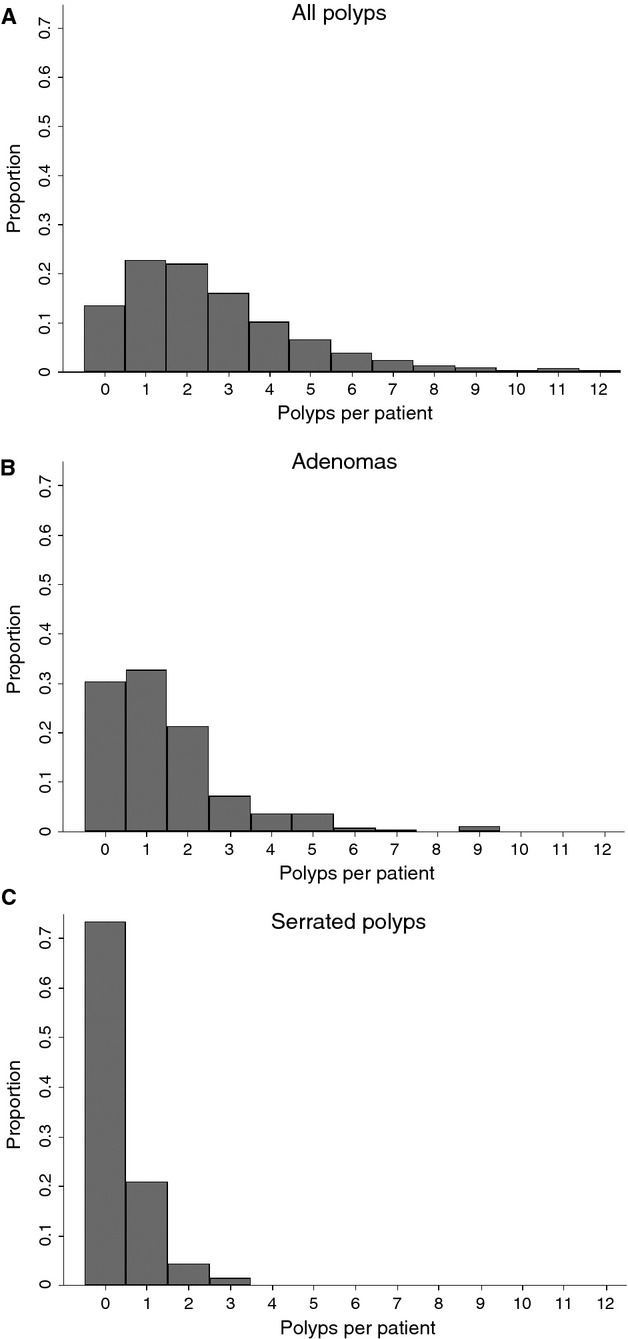
Distribution of polyps per patient for all polyps (A), adenomas (B), and serrated polyps (C).

**Figure 2:**
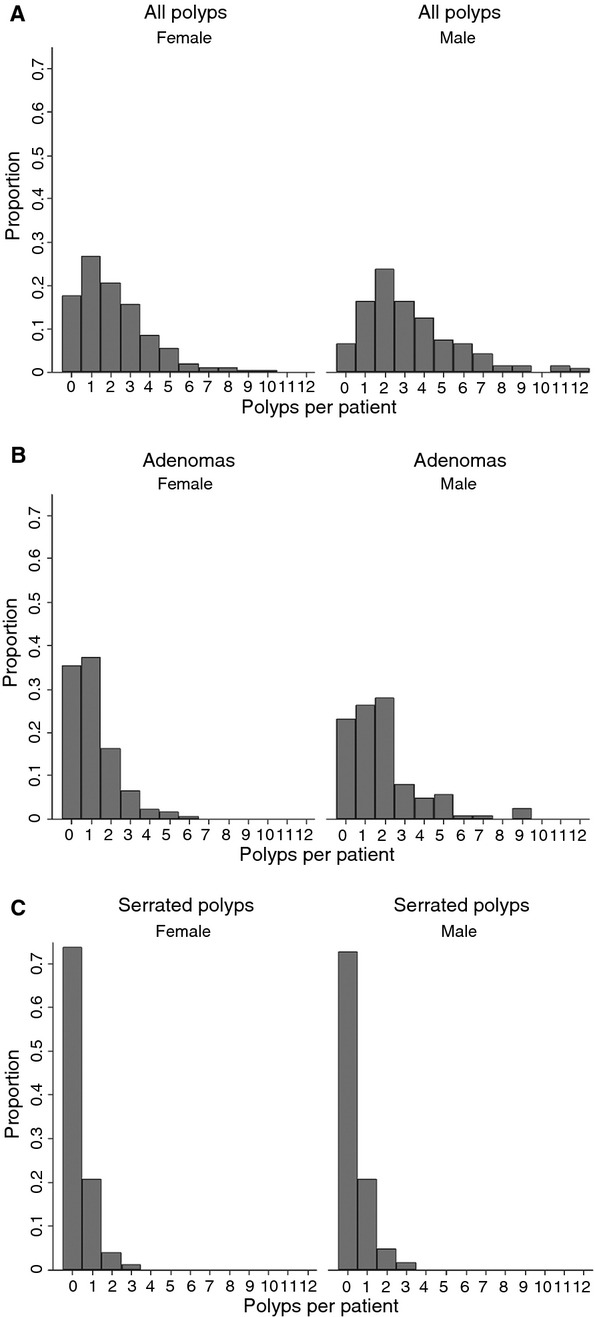
Distribution of polyps per patient for men and women for all polyps (A), adenomas (B), and serrated polyps (C).

Tables 5 and 6 present the polyps detected by their characteristics. There were 882 polyps detected among 297 (87%) of the 343 screened patients. According to their worst polyps classification, there was one patient with one cancerous polyp, 226 patients with precancerous lesions, 70 patients with polyps that were either distal hyperplastic or non-neoplastic, and 46 patients with no polyps detected. There was no overall difference between patients with a cancer history versus none (*P* = 0.98). Figure 1 presents the distribution of polyp counts overall (Fig. 1A) as well as for adenomas (Fig. 1B) and serrated lesions (Fig. 1C) per person. Overall, most patients had 1, 2, or 3 polyps and the highest number in one patient was 11 polyps. Figure 2 presents the same information separated by men and women, where the higher numbers of polyps detected in men is visible with the small proportion of men with 0 polyps and more men with two or more polyps compared to women (Fig. 2A).

**Table 5:** Polyp characteristics overall and by cancer history

	All patients		No cancer history		Cancer history	
			
Classification	Patients *N* (%)	Polyps *N*	Patients *N* (%)	Polyps *N*	Patients *N* (%)	Polyps *N*
All patients	343 (100)		179 (100)		163 (100)	
All polyps	297 (87)	882	157 (88)	454	139 (85)	416
Cancer	1 (0)	1	0 (0)	0	1 (0)	1
Precancerous polyps	226 (66)	479	118 (66)	236	107 (66)	233
Adenoma	207 (60)	422	110 (61)	212	96 (59)	201
Multiple (≥3)	47 (14)	–	22 (12)	–	24 (15)	–
Tubular	190 (55)	376	103 (58)	187	86 (53)	180
Tubulovillous	3 (1)	3	3 (2)	3	0 (0)	0
Advanced	10 (3)	13	7 (4)	9	3 (2)	4
Serrated polyps	79 (23)	100	38 (21)	46	40 (25)	53
Serrated adenoma*	36 (11)	43	21 (12)	22	15 (9)	21
Proximal hyperplastic	49 (14)	57	21 (12)	24	27 (17)	32
Advanced	4 (1)	5	3 (2)	3	1 (1)	2
Distal hyperplastic/Non-neoplastic**	70 (20)	402	39 (22)	218	31 (19)	182
No polyps found	46 (13)	–	22 (12)	–	24 (15)	–

* Patients with serrated adenomas count both in the adenoma and serrated categories.

** Patient counts include patients who had polyps, but all identified polyps were free from cancer or precancerous features. The polyp count includes all such polyps even if the patient also had precancerous or cancerous polyps.

**Table 6:** Polyp characteristics by shape

	All	0–1p *N* (%)	0–1s *N* (%)	0–IIa *N* (%)	0–IIb *N* (%)	0–IIc *N* (%)
All polyps	670	8 (100)	158 (100)	465 (100)	37 (100)	2 (100)
Location						
Right						
Appendiceal orifice	1 (0)	–	–	1 (0)	–	–
Ascending colon	119 (18)	1 (13)	33 (21)	79 (17)	6 (16)	–
Cecum	73 (11)	1 (13)	16 (10)	48 (10)	6 (16)	2 (100)
Distal transverse colon	2 (0)	–	–	–	2 (5)	–
Hepatic flexure	35 (5)	–	11 (7)	20 (4)	4 (11)	–
Ileocecal valve	8 (1)	–	–	8 (2)	–	–
Transverse colon	81 (12)	–	17 (11)	59 (13)	5 (14)	–
Left						
Descending colon	95 (14)	–	17 (11)	73 (16)	5 (14)	–
Rectosigmoid junction	14 (2)	–	5 (3)	9 (2)	–	–
Rectum	111 (17)	1 (13)	34 (22)	74 (16)	2 (5)	–
Sigmoid colon	129 (19)	5 (63)	25 (16)	92 (20)	7 (19)	–
Unspecified location	2 (0)	–	–	2 (0)	–	–
Diagnosis						
Adenoma	326 (49)	7 (88)	93 (59)	199 (43)	25 (68)	2 (100)
Serrated adenoma	32 (5)	1 (13)	3 (2)	23 (5)	5 (14)	–
Hyperplastic polyps*	45 (7)	–	5 (3)	39 (8)	1 (3)	–

558 polyps were collected prior to including Paris Classification of polyps in our endoscopy reports and are not included here. Additionally, there was 1 polyp that was 0–III in the sigmoid colon that was diagnosed as cancer. (*Hyperplastic polyps located proximal to the splenic flexure.)

### Adenoma detection

The adenoma detection rate was 60% overall and 71% in men and 53% in women. Figure 1B shows that the mean number of adenomas per patient was 1.4 (1.2, 1.6). For men and women, the mean numbers of adenomas detected were 1.9 (1.5, 2.2) and 1.1 (0.9, 1.3), respectively (*p* < 0.0001; Fig. 2B). The mean number of adenomas per patient positive for precancerous lesions (adenomas or serrated lesions) was 1.9 (1.7, 2.1).

### Serrated lesion and precancerous lesion detection

Serrated lesions were detected in 79 patients (serrated lesion detection rate: 23%), and the detection rate remained the same in men and women (Fig. 2C). Among the 100 serrated lesions diagnosed in 79 patients, 43 were serrated adenomas, and 57 were hyperplastic polyps proximal to splenic flexure. The serrated lesions were located in the cecum (*n* = 22), ascending colon (*n* = 32), transverse colon (*n* = 34), descending colon (*n* = 7), sigmoid colon (*n* = 5), and rectum (*n* = 4), respectively. Majority of the polyps were non-polypoid (36/40; the Paris Classification was used during the later part of the study).

The precancerous lesion (adenoma and serrated lesion) detection rate was 66% (men: 78% and women: 58%; *P* = 0.01). The mean number of adenomas or serrated lesions per screening colonoscopy was 1.6 (1.4, 1.8).

### Advanced adenoma and multiple adenoma detection

The advanced adenoma detection rate was 3. The mean number of adenomas in patients with advanced adenomas was 4.2 (2.2, 6.2).

The multiple adenoma (≥3 adenomas) detection rate was 14%. The detection rate of three or more adenomas or serrated lesions was 16%.

There were no statistically significant differences between the cancer and no cancer groups for adenoma (*P* = 0.63), serrated lesion (*P* = 0.47), or advanced adenoma (*P* = 0.56) or multiple adenoma (*P* = 0.51) detection rates, even though some mild trends appear in Table 6.

## Discussion

This study demonstrates that a comprehensive colonoscopy screening program designed to optimize quality parameters results in a high adenoma and serrated polyp detection rate which exceeds current quality standards.

The adenoma detection rate is the most widely used metric for assessing quality of colonoscopy screening. In our study, the comprehensive colonoscopy screening resulted in adenoma detection rate of 60%. This rate was higher than that reported in a large meta-analysis (22–58.2%) [50], chromoendoscopy studies (45–59%) [51–54], and wide-angle colonoscopy (27%) studies [55].

The comprehensive colonoscopy screening program was associated with an advanced adenoma prevalence of 3%. The prevalence of advanced adenomas reported in a large meta-analysis varied between 2.48% and 9.67% [50].

Another emerging quality indicator of screening colonoscopy is that the serrated lesion detection rate and the miss rates and variability in detection for serrated lesions are greater than those for adenomas [56, 57]. The prevalence of serrated lesions in patients undergoing comprehensive colonoscopy screening program was 23% compared to those reported in the literature 13% ± 7.8% (1–18%) [56–58], and closer to those reported in the autopsy studies (25–50%) [59–61].

The ultimate goal of screening colonoscopy is to prevent colorectal cancer by detecting and removing all precancerous lesions, not only adenomas but also serrated lesions [43]. Although the mean number of adenomas per patient provides additional information about the performance of the colonoscopist [10], reporting on precancerous lesion detection rate (adenoma + serrated lesion detection rate) and the mean number of precancerous lesions per patient may serve as a better bench mark [10]. We report 66% precancerous lesion detection rate and 1.4 precancerous lesions per patient. This could be due to the type of population screened or the impact of a comprehensive colonoscopy screening program.

We doubt that the high prevalence of adenomas and serrated lesions in our study population is due to the population selected for screening at a cancer center, because there was no difference between the cancer and no cancer groups of patients in the adenoma detection rates.

Age is the single most important independent determinant of the prevalence of adenomas, advanced adenomas, and cancer, with twofold and sevenfold higher rates, respectively, among older cohorts (≥65 years) [50]. In contrast to the advanced adenoma prevalence rates of 8.89% and 9.67% in cohorts with a mean age of 65 years and 68.6 years, respectively [62, 63], the advanced adenoma detection rate in our study population (mean age of 58.2 years) is 3%, suggesting additional benefit of a comprehensive colonoscopy screening program [26, 33].

We believe that the high adenoma detection rate as well as the high serrated lesion detection rate was due to the additive effect of multiple factors incorporated in the comprehensive colonoscopy screening protocol each of which were clearly demonstrated to improve adenoma detection, such as ensuring high-quality colon preparation [16, 34, 35], routine use of a cap fitted colonoscope to improve the examination of inter-haustral area [38, 39, 64], adequate distension of the colon with the use of patient position change as needed [37], and use of a screening protocol to detect flat lesions by a trained endoscopy team [36, 40, 65–68]. Although the importance of position of the video monitor and slight overcorrection of the eye glasses for better visual acuity has not been formally addressed, the role of gaze pattern on adenoma detection has been found to improve adenoma detection [69]. In addition, recording the videos of subtle flat lesions routinely as part of our practice and reviewing them may help train the eye for pattern recognition of serrated lesions.

An important strength of the study is the use of a comprehensive colonoscopy screening program in all patients undergoing their first exam, with over 98% cecal intubation, excellent quality of bowel cleansing (Boston Bowel Preparation Scale of 8.97), examination by an endoscopy team trained in the detection of subtle flat lesions, and recording of the data based on the recommendations of the Quality Assurance Task Group of the National Colorectal Cancer Roundtable [9, 70, 71].

There are several limitations to this study that need to be emphasized. A major drawback is that this report is the experience of a single endoscopist. Whether or not various measures utilized in the protocol based on the ASGE/American College of Gastroenterology task force recommendations to improve the quality of colonoscopy could be widely adopted is not clear, given the structure of pay for procedure modus of operation in the United States [72]. However, the Institute of Medicine's initiative in improving the quality of care in the United States is an opportunity to implement several strategies similar to those implemented in the United Kingdom – ensuring colonoscopists meet high standard before starting screening and through ongoing quality assurance [10, 73]. Another drawback was the retrospective nature of the study; however, the detailed endoscopy data recording minimizes some of the drawbacks of a retrospective study [41, 49]. The total duration of the colonoscope insertion and withdrawal in our study may be considered impractical in community practice by some practitioners. Several factors account for the prolonged duration of procedure in this study, such as the use of conscious sedation of patients, routine use of simethicone flush to clear the bubbles followed by suction of all the luminal contents of the colon during endoscope insertion for better examination of a dry clean colon during the withdrawal, removal of polyps during insertion, higher adenoma detection rate, higher number of polyps removed per patient, routine use of endoscopic mucosal resection (EMR) of all flat lesions larger than 5 mm (as margins of serrated lesions are hard to define without submucosal injection of Indigo Carmine), and clearance of all polyps including EMR of large and giant lesions in the same endoscopy session.

Although the adenoma detection rate is an independent predictor of the risk of interval cancer [6], whether or not the higher adenoma detection rate observed after comprehensive colonoscopy screening protocol has any clinical benefit on limiting interval cancers or is a function of lead-time or length bias needs to be defined by long-term cohort studies. The paper by Kaminski et al. [6] does not support the notion that there is a plateau to the benefit of adenoma detection. We believe that high adenoma detection as noted in the study may have a protective effect based on a personal audit of the author's interval colorectal cancer development at another institution [74].

In summary, the comprehensive colonoscopy screening program results in high-quality screening of adenomas, advanced adenomas, serrated lesions, and multiple adenomas. The applicability of this program in improving adenoma detection across the nation needs further investigation. In the interim, many aspects of a comprehensive colonoscopy program can be readily adopted and include adequacy of the preparation, use of a cap on the endoscope, positioning of the monitor, and establishing a credentialing or continuing education related to polyp identification for all physicians and support staff involved in the performance of colonoscopy, and sufficient time for instrument withdrawal. These measures are not projected to encumber cost or insert inconvenience when performing procedures and can be differentiated from other aspects that are a function of quality assurance monitoring and documentation.

## Supporting Information

**Video S1.** Colon preparation education hyperlink (http://www3.mdanderson.org/streams/FullVideoPlayer.cfm?xml=patientEd%2Fconfig%2Fmda_Colon4--cfg).

**Video S2.** http://www.youtube.com/user/GottumukkalaSRaju?feature=guide.
