# Supplementary material for: Adenoma detection in patients undergoing a comprehensive colonoscopy screening
Source: Cancer Med. 2013 Apr 20;2(3):391–402. doi: 10.1002/cam4.73 (PMC3699850; doi:10.1002/cam4.73)
Supplement: Supplementary file 1 [file cam40002-0391-SD1.docx]

**Video S1.** Colon preparation education hyperlink (http://www3.mdanderson.org/streams/FullVideoPlayer.cfm?xml=patientEd%2Fconfig%2Fmda_Colon4--cfg).
